# Infections Caused by Antimicrobial Drug-Resistant Saprophytic Gram-Negative Bacteria in the Environment

**DOI:** 10.3389/fmed.2017.00183

**Published:** 2017-10-30

**Authors:** Eva Raphael, Lee W. Riley

**Affiliations:** ^1^Division of Infectious Diseases and Vaccinology, School of Public Health, University of California, Berkeley, Berkeley, CA, United States

**Keywords:** Gram-negative bacteria, saprophytes, drug-resistant Gram-negative bacteria, *Enterobacter aerogenes*, *Pantoea agglomerans*, *Pseudomonas putida*

## Abstract

**Background:**

Drug-resistance genes found in human bacterial pathogens are increasingly recognized in saprophytic Gram-negative bacteria (GNB) from environmental sources. The clinical implication of such environmental GNBs is unknown.

**Objectives:**

We conducted a systematic review to determine how often such saprophytic GNBs cause human infections.

**Methods:**

We queried PubMed for articles published in English, Spanish, and French between January 2006 and July 2014 for 20 common environmental saprophytic GNB species, using search terms “infections,” “human infections,” “hospital infection.” We analyzed 251 of 1,275 non-duplicate publications that satisfied our selection criteria. Saprophytes implicated in blood stream infection (BSI), urinary tract infection (UTI), skin and soft tissue infection (SSTI), post-surgical infection (PSI), osteomyelitis (Osteo), and pneumonia (PNA) were quantitatively assessed.

**Results:**

Thirteen of the 20 queried GNB saprophytic species were implicated in 674 distinct infection episodes from 45 countries. The most common species included *Enterobacter aerogenes, Pantoea agglomerans*, and *Pseudomonas putida*. Of these infections, 443 (66%) had BSI, 48 (7%) had SSTI, 36 (5%) had UTI, 28 (4%) had PSI, 21 (3%) had PNA, 16 (3%) had Osteo, and 82 (12%) had other infections. Nearly all infections occurred in subjects with comorbidities. Resistant strains harbored extended-spectrum beta-lactamase (ESBL), carbapenemase, and metallo-β-lactamase genes recognized in human pathogens.

**Conclusion:**

These observations show that saprophytic GNB organisms that harbor recognized drug-resistance genes cause a wide spectrum of infections, especially as opportunistic pathogens. Such GNB saprophytes may become increasingly more common in healthcare settings, as has already been observed with other environmental GNBs such as *Acinetobacter baumannii* and *Pseudomonas aeruginosa*.

## Introduction

In 2013, the Centers for Disease Control and Prevention released a report “Antibiotic Resistance in the United States, 2013” that classified groups of drug-resistant microorganisms into “urgent threat,” “serious threat,” and “concerning threat” pathogens (1). Drug-resistant Gram-negative bacterial (GNB) pathogens comprised 2 of the 3 “urgent threat” pathogens and 7 of 12 “serious threat” pathogens (1). GNBs cause about one-third of healthcare-associated (HCA) infections in the United States, and the proportion of multidrug-resistant and non-fermentative organisms (*Pseudomonas aeruginosa, Acinetobacter baumannii*) causing these infections has increased dramatically in the last 20 years, according to the National Healthcare Safety Network (NHSN) surveys (2). While still relatively low in most hospitals in the United States, multidrug-resistant *Acinetobacter baumannii*, an environmental saprophyte, has become endemic in healthcare settings in several Latin American countries, and has surpassed *P. aeruginosa* as the most common non-fermentative GNB in such settings (3). The progressive increase in drug resistance and new species of GNBs causing HCA infections may represent an evolution in drug-resistant GNB infections that may profoundly affect the future landscape of epidemiology and clinical management of such infections in the healthcare environment globally.

Most of the new antimicrobial drug-resistance mechanisms discovered during the last 20 years have been those found in GNBs (4–11). In 2011, Raphael et al. reported identification of a variety of saprophytic GNB species on retail spinach that were resistant to antimicrobial agents commonly used in clinical settings (12). Twelve of 20 species found on spinach expressed extended-spectrum β-lactamases (ESBLs), based on their resistance to ceftazidime and cefotaxime (12). Two strains of *Pseudomonas putida* and one strain of *Pseudomonas teessidea* contained *bla*_CTX-M-15_, the most common ESBL gene distributed globally and carried frequently by the most common pandemic extraintestinal pathogenic *E. coli* (ExPEC) lineage ST131 (13–17). They suggested that environmental saprophytes may serve as a reservoir for some of the common drug-resistance genes found in human GNB pathogens (12).

Saprophytes are environmental microorganisms that survive on dead organic matter. While no pathogenic GNB organisms were found on spinach in the above study, we were concerned that a large proportion of these saprophytic organisms were drug resistant. We wished to know whether saprophytic GNBs that carry recognized drug-resistance genes cause human infections. After all, pathogenic GNBs, such as *K. pneumoniae, P. aeruginosa*, and *Acinetobacter* spp., are environmental GNB organisms. Here, we conducted a systematic bibliographic search of human infections caused by well-recognized environmental saprophytic GNB species. We present our review results and suggest that these saprophytic GNB may represent a harbinger of the next phase of the evolution of GNB infections, especially in healthcare settings.

## Methods

### Data Sources and Searches

**Figure 1:**
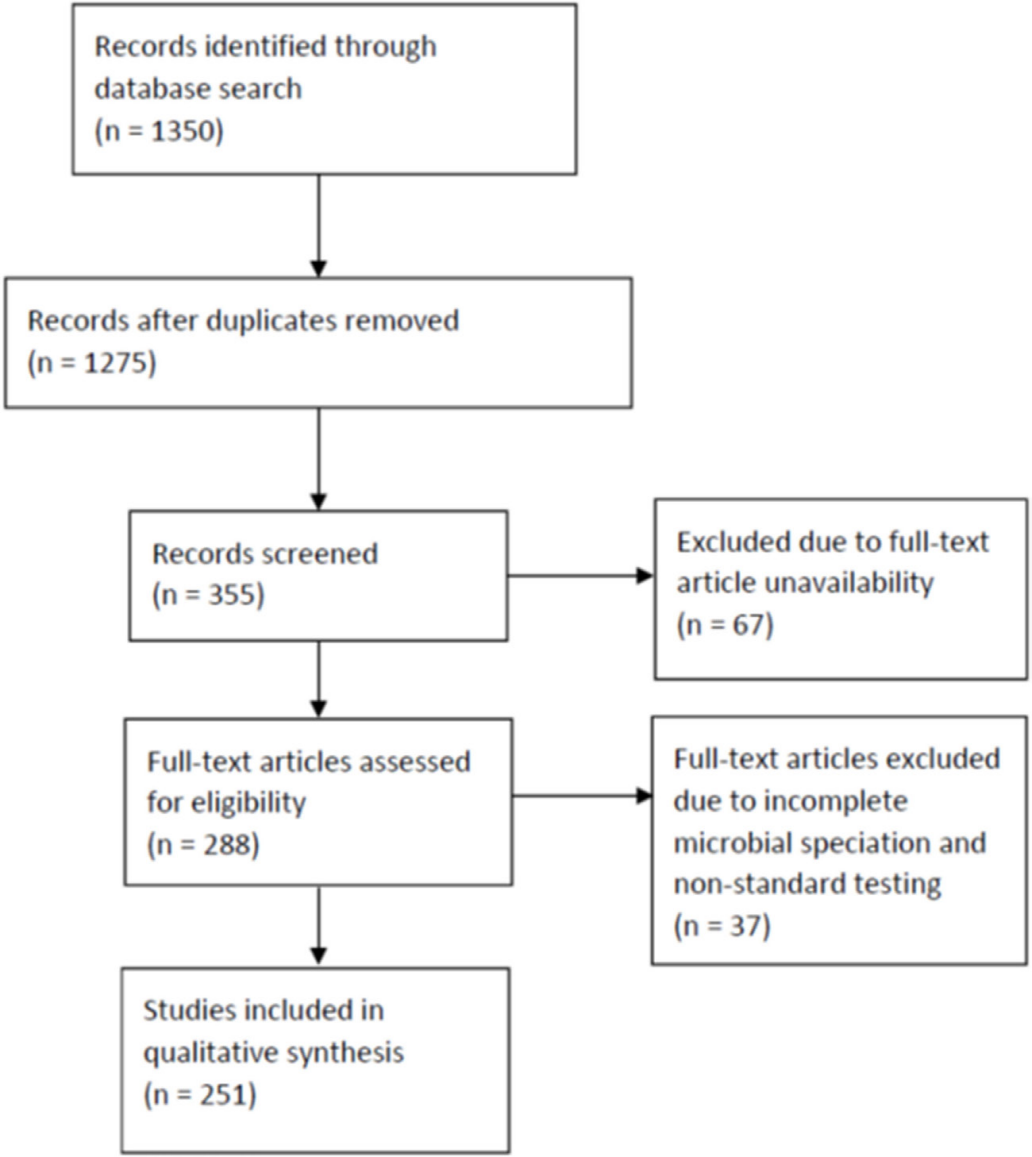
Preferred Reporting Items for Systematic Reviews and Meta-Analyses flow diagram for searched articles.

We followed the guideline outlined in the Preferred Reporting Items for Systematic Reviews and Meta-Analyses (PRISMA) to conduct this review (Figure 1). Using the names of GNB species listed in Table 1 as search terms, we queried PubMed for abstracts and full-length articles that were published in English, French, and Spanish between January 2006 and July 2014. The selected GNB species were based on those frequently found on retail spinach in a previous report (1). If more than 200 articles appeared for one species, the term “infections” was added to the search. In certain cases, the term “human infections” or “hospital infection” was added. If an organism was not reported as a cause of human infection after 2006, the search was widened to include articles published before 2006.

**Table 1:** Case reports and hospital microbiological surveys reporting infections caused by saprophytic Gram-negative bacteria (GNB) species previously identified on retail spinach [Raphael et al (12)].

Organism	Case reports (cases)	Hospital-based microbiological surveys (isolates)	Countries reporting infection	Reference
*Acinetobacter rhizosphaerae*	0	0		

*Enterobacter aerogenes/Klebsiella aerogenes*	24 (27)	87 (7,498)	Japan, Nigeria, US, Belgium, Taiwan, Italy, China, Korea, Portugal, France, Sri Lanka, Spain, Belgium, Switzerland, UK, Indonesia, Brazil, India, Greece, Saudi Arabia, Canada, Philippines, Austria, Costa Rica, Bulgaria, Kazakhstan, Turkey, Fiji, Ivory Coast, Netherlands, Australia, Gran Canaria, Nepal, Nigeria, Venezuela, Germany	(13, 18–128)

*Enterobacter amnigenus*	6 (8)	2 (6)	China, India, US, Italy, France, Spain	(105, 129–136)

*Enterobacter asburiae*	2 (2)	9 (108)	US, Korea, Switzerland, Norway, Portugal, Germany, Nigeria	(33, 47, 68, 82, 137–143)

*Enterobacter kobei*	1 (1)	3 (20)	Germany, Poland, Switzerland, Japan	(33, 144–147)

*Enterobacter ludwigii*	1 (1)	1 (3)	India, Germany	(144, 145, 148)

*Erwinia persicina/persicinus*	1 (1)	0	US	(149)

*Erwinia rhapontici*	0	0		

*Pantoea agglomerans/Enterobacter agglomerans*	37 (56)	13 (154)	Spain, US, Belgium, Taiwan, Italy, Kuwait, Korea, France, UK, Netherlands, India, Israel, Canada, Germany, Turkey, Brazil, Zimbabwe, Mexico, Bulgaria, Nigeria, Greece, Malaysia	(6, 47, 58, 63, 70, 79, 150–191)

*Pantoea ananatis*	2 (2)	2 (8)	Tunisia, India, Belgium, Georgia	(192–195)

*Pseudomonas fragi*	0	0		

*Pseudomonas libanensis*	0	0		

*Pseudomonas orientalis*	0	0		

*Pseudomonas putida*	13 (29)	19 (176)	Japan, Spain, Iraq, US, Turkey, US–Hawaii, UK, Brazil, Poland, Taiwan, Italy, Belgium, Korea, France, Argentina, China, Canary islands	(59, 189, 196–225)

*Pseudomonas reactans*	0	0		

*Pseudomonas rhodesiae*	0	0		

*Pseudomonas teessidea*	0	0		

*Pseudomonas syringae*	0	0		

*Rahnella aquatilis*	13 (15)	1 (1)	France, Spain, Japan, Switzerland, Greece, Germany, Saudi Arabia, US, Korea, Italy, Belgium	(183, 224, 226–237)

*Rhizobium* spp.	21 (24)	0	UK, Turkey, India, Japan, Belgium, US, Greece, Kuwait, Colombia, France, Taiwan, Italy, Venezuela, Spain, Portugal	(90, 238–257)

*Serratia fonticola*	4 (4)	1 (1)	France, Canada, Switzerland	(133, 258–261)

*Serratia proteamaculans*	2 (5)	0	France, US	(262, 263)

### Study Selection

We first identified 1,350 abstracts. After duplicates were removed, we reviewed 1,275 abstracts (Figure 1). We excluded for analysis abstracts, as well as reviews and microbiology papers that did not include human clinical data, or articles that described only episodes of colonization, laboratory contamination, or pseudo-infection (e.g., line or catheter contamination). Full-text articles were not available for 67 of these abstracts. We identified 355 abstracts after exclusion and obtained full-text articles for 288 of them. Of the full-text articles, 37 were excluded further due to incomplete GNB speciation and non-standard microbiologic test procedures reported in the articles. We, thus, included 251 articles for this review (18–76, 78–103, 105–245, 247–246); of these, 124 were case series reports and 127 were hospital microbiologic survey reports.

### Data Extraction

We analyzed the selected articles for the GNB species implicated as causative agents of infections that were most commonly reported among the articles—blood stream infection (BSI), urinary tract infection (UTI), skin and soft tissue infection (SSTI), post-surgical infection (PSI), osteomyelitis (Osteo), and pneumonia (PNA). Other reported infections were grouped under the “other” category. The reports were also reviewed for descriptions of mortality, comorbidity, as well as healthcare interventions associated with the above infectious disease episodes. Finally, we reviewed microbiologic reports that accompanied these articles for drug-susceptibility test results as well as genetic analyses of drug-resistance determinants.

### Quality Assessment

To assure search completeness and reproducibility of our findings, we conducted the same search described above on three different occasions—in 2011, 2013, and 2015. We included articles only from peer-reviewed journals. We reviewed in detail the descriptions of microbiologic test procedures used to detect and identify the implicated GNB species. We also examined the descriptions of drug-susceptibility test procedures to ascertain their standardization. Both authors ER and LR independently reviewed the quantitative data analysis results obtained from examination of these 251 articles.

### Data Synthesis and Analysis

We first quantified the type and number of GNB species implicated in infections reported in the 251 selected articles. We then examined the frequencies of GNB species associated with the six most common infections described in the articles, separated by case series and hospital survey studies, reported from different regions of the world. We compared mortality and comorbidities associated with each of these infections and assessed unusual events that were described to trigger these infectious disease episodes. We then analyzed the microbiologic data, which included antimicrobial drug-susceptibility test results and PCR-based and nucleic acid sequence-based detection of drug-resistance genes.

## Results

A previous study identified 20 distinct saprophytic GNB species among 231 randomly selected colonies cultured on MacConkey agar plates from 25 batches of retail spinach rinsates (12). Here, these 20 species were queried in the bibliographic search engines. Thirteen of these species were reported to cause human infections in the reviewed literature (Table 1). We identified 175 cases described in 124 separate case report series. From 127 hospital microbiology surveys, 7,671 isolates from infected subjects were reported. These infections were reported from 48 countries. The most commonly reported GNB species was *Enterobacter aerogenes. E. aerogenes* together with *Pantoea agglomerans* (formerly *Enterobacter agglomerans*) and *Pseudomonas putida* together comprised more than half of the reported cases of infection caused by these saprophytic GNBs.

The most commonly isolated GNB species reported from 127 hospital-based microbiologic surveys was also *Enterobacter aerogenes*; 7,498 *E. aerogenes* isolates from 87 separate hospital surveys were reported (Table 1). In both case series and hospital survey reports, the most common GNB species were identical—*E. aerogenes, P. putida, P. agglomerans*, and *E. asburiae*.

The bibliographic search identified 674 individuals who were diagnosed with infection caused by the 13 GNB species. These saprophytes caused a wide spectrum of infections (Table 2). They were isolated from 443 patients with BSI, 48 patients with SSTI, 36 patients with UTI, 28 patients with PSI, 21 patients with PNA, 16 patients with Osteo, and 82 with other infections. In addition to blood, urine, skin and soft tissue, surgical wounds, bone, and lungs, these GNB species were isolated from many other body sites such as the peritoneum, eyes, and the central nervous system (Table S1 in Supplemental Material).

**Table 2:** Infections caused by saprophytic Gram-negative bacteria organisms.

Organism	Number of cases (deaths, cases with no underlying medical condition)						
	BSI	UTI	PSI	SSTI	Osteo	PNA	Other
*Enterobacter aerogenes*	232 (28, 0)	7 (1, 0)	3 (0, 0)	6 (1, 1)	3 (1, 1)	1 (0, 0)	17 (1, 1)
*Enterobacter amnigenus*	3 (2, 0)	0	0	1 (0, 1)	0	0	4 (0, 1)
*Enterobacter asburiae*	16 (0, 0)	17 (0, 0)	3 (0, 0)	10 (0, 0)	0	10 (0, 0)	5 (0, 0)
*Enterobacter kobei*	1 (0, 0)	1 (0, 0)	1 (0, 0)	0	0	0	0
*Enterobacter ludwigii*	0	1 (0, 0)	1 (0, 0)	0	0	0	1 (0, 0)
*Erwinia persicina*	0	1 (0, 0)	0	0	0	0	0
*Pantoea agglomerans*	67 (12, 1)	4 (0, 0)	0	25 (0, 2)	12 (0, 1)	2 (0, 0)	30 (1, 3)
*Pantoea ananatis*	1 (0, 0)	0	0	0	0	0	2 (0, 1)
*Pseudomonas putida*	98 (14, 1)	3 (0, 1)	15 (4, 1)	3 (1, 1)	0	7 (2, 0)	6 (0, 2)
*Rahnella aquatilis*	10 (0, 1)	2 (0, 0)	3 (0, 0)	0	0	0	3 (0, 1)
*Rhizobium* spp.	14 (0, 1)	0	2 (0, 0)	0	1 (0, 0)	0	9 (0, 1)
*Serratia fonticola*	1 (0, 0)	0	0	3 (0, 1)	0	0	1 (0, 0)
*Serratia proteamaculans*	0	0	0	0	0	1 (1, 0)	4 (0, 0)
Total	443 (56, 4)	36 (1, 1)	28 (4, 1)	48 (2, 6)	16 (2, 1)	21 (3, 0)	82 (2, 10)

*BSI, blood stream infection; UTI, urinary tract infection; PSI, post-surgical infection; SSTI, skin and soft tissue infection; Osteo, osteomyelitis; PNA, pneumonia*.

The most frequently isolated GNB species cultured from blood were *E. aerogenes* (232 cases), *P. putida* (98 cases), and *P. agglomerans* (67 cases). They were responsible for 89% of the BSI cases caused by the 13 saprophytic GNB species. Mortality from BSI associated with these organisms was 12, 14, and 18%, respectively. All of the patients with BSI, except 4, had comorbidities or underlying medical problems (Table 3 and supplemental table). The most common comorbidities associated with *E. aerogenes* BSI were cancer (35 cases), biliary disease (21 cases), acute respiratory distress syndrome (ARDS) (10 cases), premature birth (10 cases), and diabetes (9 cases). Many of these patients had an invasive procedure, chemotherapy, or immunosuppressive drugs within 14 days of the bacteremia. Of these BSI cases, 49 were reported as nosocomial infections.

**Table 3:** Comorbidities associated with infections caused by saprophytic Gram-negative bacteria species.

Organism	Infection	CVD	Pulmonary	GI	DM-Endocrine	GU	OB-GYN	Genetic	Rheumatologic	Neurologic	Heme-Oncologic	Immunologic	MSK	Neonatal disease	Psych and PSA	Other infections
*Enterobacter aerogenes*	Blood stream infection (BSI)	✓	✓	✓	✓	✓				✓	✓	✓		✓	✓	✓
	Urinary tract infection (UTI)	✓	✓	✓	✓	✓		✓				✓				✓
	Post-surgical infection (PSI)															
	Skin and soft tissue infection (SSTI)				✓	✓					✓	✓				
	Osteomyelitis (Osteo)				✓						✓					
	Pneumonia (PNA)					✓										✓
	Others	✓		✓	✓	✓	✓	✓				✓		✓	✓	✓

*Enterobacter amnigenus*	BSI	✓									✓	✓				
	SSTI															
	Others	✓			✓											✓

*Enterobacter asburiae*	BSI	✓									✓	✓		✓		
	UTI					✓					✓					
	PSI															
	SSTI															✓
	PNA											✓				
	Others															

*Enterobacter kobei*	BSI						✓				✓					
	UTI						✓				✓					
	PSI						✓				✓					

*Enterobacter ludwigii*	UTI															
	PSI												✓			
	Others															

*Erwinia persicina*	UTI	✓			✓						✓					

*Pantoea/Enterobacter agglomerans*	BSI	✓	✓	✓	✓	✓	✓	✓		✓	✓	✓		✓		✓
	UTI	✓	✓	✓				✓		✓	✓					
	PSI															
	SSTI	✓	✓	✓				✓		✓	✓					
	Osteo	✓	✓	✓						✓	✓					
	PNA	✓		✓	✓			✓			✓	✓				✓
	Others	✓	✓	✓	✓	✓		✓		✓	✓	✓	✓		✓	✓

*Pantoea ananatis*	BSI	✓		✓	✓					✓	✓					
	Others								✓							✓

*Pseudomonas putida*	BSI	✓	✓	✓	✓	✓	✓		✓	✓	✓	✓		✓		✓
	UTI			✓			✓				✓					
	PSI	✓	✓	✓		✓					✓					✓
	SSTI	✓				✓					✓	✓				✓
	PNA	✓	✓	✓	✓					✓	✓	✓				
	Others					✓					✓	✓				✓

*Rahnella aquatilis*	BSI		✓	✓	✓	✓			✓		✓	✓			✓	✓
	UTI	✓			✓	✓						✓				✓
	PSI				✓	✓					✓		✓			
	Others	✓										✓				✓

*Rhizobium* spp.	BSI	✓	✓	✓	✓	✓	✓			✓	✓	✓		✓		
	PSI	✓		✓	✓							✓				
	Osteo				✓											
	Others	✓		✓	✓	✓		✓		✓		✓				✓

*Serratia fonticola*	BSI												✓			
	SSTI												✓			
	Others															

*Serratia proteamaculans*	PNA			✓								✓				
	Others												✓			

*CVD, cardiovascular disease; GI, gastrointestinal disease; DM, diabetes mellitus; GU, genitourinary disease; OB-GYN, obstetrics-gynecology disease; MSK, musculoskeletal disease; PSA, polysubstance abuse*.

The most common comorbidity associated with *P. putida* BSI were solid (31 cases) and hematologic (7 cases) malignancies (Table 3 and supplemental table). Contaminated central venous catheter (CVC), catheter lock infused with heparin, and other short-term intravascular devices were implicated with BSI in a large proportion of these patients (Table 4 and supplemental table).

**Table 4:** Healthcare-associated interventions associated with infections caused by saprophytic Gram-negative bacteria species.

Organism	Infection	Ortho/trauma surgery	Interventional cardiologic procedures	Transplant	Neurosurgery	GI procedures	Other surgery	Chemotherapy	Central venous catheter	CCPD/HD	Foley catheter	Mechanical ventilation	Transfusion/infusion
*Enterobacter aerogenes*	Blood stream infection (BSI)	✓	✓	✓		✓		✓	✓				
	Urinary tract infection (UTI)										✓		
	Post-surgical infection (PSI)				✓		✓						
	Skin and soft tissue infection (SSTI)	✓								✓			
	Osteomyelitis (Osteo)	✓											
	Pneumonia (PNA)									✓			
	Others		✓		✓		✓						

*Enterobacter amnigenus*	BSI								✓				✓

*Enterobacter asburiae*	PSI					✓	✓						

*Enterobacter kobei*	BSI						✓						
	UTI						✓						
	PSI						✓						

*Pantoea/Enterobacter agglomerans*	BSI						✓		✓	✓		✓	✓
	Osteo	✓											
	PNA			✓									
	Others	✓							✓	✓			

*Pantoea ananatis*	BSI					✓							
*Pseudomonas putida*	BSI	✓		✓		✓	✓	✓	✓		✓	✓	✓
	UTI					✓	✓				✓		
	PSI	✓		✓		✓	✓			✓			
	SSTI	✓					✓						
	PNA					✓	✓					✓	
	Others			✓		✓	✓						

*Rahnella aquatilis*	BSI					✓		✓	✓				✓
	UTI			✓									
	PSI					✓	✓						✓

*Rhizobium* spp.	BSI	✓	✓	✓		✓		✓	✓	✓		✓	
	PSI		✓				✓						
	Others						✓		✓	✓	✓		

*Serratia fonticola*	BSI	✓											
	SSTI	✓											

*Serratia proteamaculans*	PNA					✓	✓						
	Others	✓											

Patients with *P. agglomerans* BSI also had a wide variety of underlying medical conditions and comorbidities, but no single group of comorbidity, such as cancer, was predominant, as they were with *E. aerogenes* and *P. putida* BSI (Table 3 and supplemental table). Contaminated CVCs, however, were commonly associated with BSI in this group also (Table 4 and supplemental table).

Interestingly, the frequency of types of infection varied according to GNB species. As described above, more than 80% of BSIs were caused by just three of the saprophytic GNBs—*E. aerogenes, P. putida*, and *P. agglomerans*. On the other hand, just two species—*P. agglomerans* (25 cases) and *Enterobacter asburiae* (10 cases)—accounted for 73% of the 48 SSTI cases. More than half of the 36 UTI cases were caused by *E. asburiae*. *E. asburiae* (10 cases) and *P. putida* (7 cases) were associated with 81% of the 21 PNA cases, while *P. putida* accounted for 54% of the 28 PSI patients. The most commonly reported species *E. aerogenes* was not associated with any PNAs and it was isolated from only three cases of PSI and from seven UTI cases.

A few of these infections were triggered by accidental or atypical events (Table 5). *E. amnigenus, E. asburiae and P. putida, P. agglomerans, Rhizobium* spp., *and S. fonticola* infections were reported from six individuals involved in motor vehicle accidents (MVA) (129, 133, 143, 200, 249, 260). Three cases of *P. agglomerans* infections were attributed to (1) Hickman catheter contaminated with water from botanical garden (160), (2) puncture wound involving date palm tree thorn (70, 167), and (3) plant-product splinters (70, 167). One case of *P. ananatis* infection followed an ocular trauma with a rice husk (193). A case of *Serratia fonticola* infection resulted from a bear bite (258) (Table 5).

**Table 5:** Unusual events leading to infection by saprophytic Gram-negative bacteria species.

Organism	Infection	Unusual event
*Enterobacter amnigenus*	Skin and soft tissue infection (SSTI)	Motor vehicle accident (MVA)

*Enterobacter asburiae*	SSTI	All-terrain vehicle crash

*Pantoea/Enterobacter agglomerans*	Blood stream infection (BSI)	Hickman catheter contaminatd with water from botanical garden
	SSTI	Puncture wound in foot, foreign body in wound (wood splinter, date palm thorn)
	Others	Plant-product puncture (rose thorn, date palm tree thorn, wood splinters, lemon tree thorn, wood fence splinter), contamination of catheter with non-sterile surface, worked in garden

*Pantoea ananatis*	Others	Ocular trauma with rice husk

*Pseudomonas putida*	BSI	MVA
	Post-surgical infection (PSI)	Blast injuries, MVA with esophageal perforation
	SSTI	Blast injuries
	Others	Fall

*Rahnella aquatilis*	BSI	IV injection of contaminated D5W + Vit B complex outside hospital

*Rhizobium* spp.	BSI	Contamination of CVC line due to exposure to soil, MVA
	Others	Perforated duodenal ulcer

*Serratia fonticola*	BSI	MVA
	SSTI	Bear bite, MVA
	Others	Thorn penetration

The isolates from the reported human infections exhibited a large repertoire of antimicrobial drug resistance (Table 6). In addition to resistance to earlier generation beta-lactam drugs, there were *E. aerogenes, E. amnigenus, E. ludwigii*, and *P. agglomerans* strains that were resistant to extended-spectrum beta-lactam drugs mediated by CTX-M, TEM, SHV, and OXA. Some of the *E. aerogenes, E. amnigenus, E. asburiae*, and *P. putida* strains expressed carbapenem resistance encoded by *bla*_KPC_ and *bla*_VIM_. *E. aerogenes* and *P. putida* were the only GNB species that expressed IMP-1, a metallo-beta-lactamase, first described in *Serratia marcescens* in Japan (9), also found in *Pseudomonas aeruginosa* causing human infections (11).

**Table 6:** Drug-resistance and resistance genes of saprophytic Gram-negative bacteria species causing infection.

Organism	Fluoroquinolones	Aminoglycosides	Early generation cephalosporins	Broad-spectrum cephalosporins	Early generation penicillins	Anti-pseudomonal penicillins	Trimethoprim-sulfamethoxazole	Tetracyclines	Macrolides	Metallo-beta-lactams	Others	Drug-resistance genes
*Enterobacter aerogenes*	✓	✓	✓	✓	✓	✓	✓	✓	✓	✓	✓	bla-TEM: TEM-1, TEM-3, TEM-20, TEM-24, TEM-116 bla-SHV: SHV-4, SHV-5, SHV-12, SHV-154 bla-CTX-M: CTX-M-1, CTX-M-2, CTX-M-3, CTX-M-9, CTX-M-14, CTX-M-15, CTX-M-24, CTX-M-59, CTX-M-group 1, CTX-M-group 9, bla-OXA: OXA-1, OXA-48 bla-VIM: VIM-1, VIM-2 bla-DHA-1, bla-IMP-1 bla-KPC: KPC-2, KPC-3 AmpC qnrB7-like, intI1, intl3, qnrS, tet(A) Class 1 integron, qacDE, sul1, qnrs-1, aac(6′)-Ib, qnrA1, qnrB4, qnrB6, qnrB8, aph(3)-Ia, sul3, dfrA12, aac(6′)-Ib-cr, aacA4–catB8–aadA1, ant(3″)-Ih–aac(6)-Iid–catB8, dfrA12–orfF–aadA2, dfrA5, ramA, oqxAB, efflux pump (AcrA, TolC, OmpA, OmpX, OmpE36, PAβN), chloramphenicol efflux pump

*Enterobacter amnigenus*	✓	✓	✓		✓			✓		✓	✓	bla-KPC-2, bla-TEM-1, bla-SHV-12, rmtB bla-CTX-M: CTX-M-3, CTX-M-14

*Enterobacter asburiae*		✓	✓	✓	✓	✓		✓	✓	✓	✓	bla-KPC-2, bla-TEM-1, ARC-4, dfrA1, dfrA14, tet(A) Class 1 and 2 integrons, qacDE, sul1

*Enterobacter kobei*			✓		✓	✓						

*Enterobacter ludwigii*	✓	✓	✓	✓	✓	✓	✓			✓		bla-OXA: OXA-2, OXA-48, CTX-M-15

*Pantoea/Enterobacter agglomerans*	✓	✓	✓	✓	✓	✓	✓	✓			✓	CTX-M-15, TEM-1, dfrA14, tet(K), qnrB1, aac-(69)-lb-cr, class 1 and 2 integron, aph, aadA1, cat1, qacDE, sul1

*Pantoea ananatis*												

*Pseudomonas putida*	✓	✓	✓	✓	✓	✓	✓		✓	✓	✓	bla-OXA: OXA-2, OXA-10 bla-VIM: VIM-1, VIM-2, VIM-4, VIM-5 bla-KPC: KPC-2 bla-IMP: IMP-1, IMP-9, IMP-12, IMP-15, IMP-16 bla-PER blaPSE VEB-1-like plasmids, class 1 integron, class 3 integron, Tn402-like transposon, aacA4, orfD, qacEΔ1/sul, aadA1, aacA7, qacEΔ1, aphA15, arr-6 (rifampin), aadB, aac6, aadA2

*Rahnella aquatilis*			✓		✓	✓	✓					bla-RHAN-1

*Rhizobium*	✓	✓		✓	✓	✓	✓				✓	

*Serratia fonticola*			✓		✓						✓	

*Serratia proteamaculans*			✓		✓			✓	✓		✓	

In addition to expressing beta-lactamases, these GNB species expressed resistance to many other classes of antimicrobial agents, including trimethoprim-sulfamethoxazole, aminoglycosides, fluoroquinolones, polymyxin, tetracyclines, rifampicin, macrolides, and chloramphenicol. Many of these resistance phenotypes were encoded by genes on mobile elements, including plasmids and integrons (Table 6).

Interestingly, some types of drug resistance co-segregated with GNB species. No ESBLs (CTX-M, SHV, TEM) were reported to be expressed by any of the *P. putida* strains, while few species other than *P. putida* expressed IMP metallo-beta-lactamases.

## Discussion

The bibliographic search covering a period from the 1990s to 2014 revealed that human infections caused by saprophytic GNB found in the environment are not uncommon. Of course, exact sources of these saprophytes that caused the infections cannot be garnered from this type of review. These 20 GNB species were targeted for this review because they were frequently found on retail spinach, which are exposed to such species environmentally.

This search most likely underestimates the true incidence since such infections are not routinely and easily diagnosed in most hospitals. An isolation of a saprophyte from a clinical sample may be discounted as a contaminant. Nevertheless, the analyses of the reports reveal several common features of these infections: (1) they are distributed worldwide, (2) they occur almost exclusively in people with underlying comorbidities or HCA interventions who come into contact with environmental sources, (3) each GNB species has a distinct predilection for a site of infection, (4) they exhibit a wide spectrum of antimicrobial drug resistance, (5) they harbor drug-resistance genes identical to those commonly carried by recognized or primary human bacterial pathogens, and (6) drug-resistance gene types co-segregate with specific GNB saprophytic species.

Both case series and hospital microbiology surveys have shown that the most commonly reported GNB species was *Enterobacter aerogenes* (Table 1). It was most frequently associated with BSI. Its high frequency worldwide could classify this bacterial species as an opportunistic pathogen rather than a saprophyte, similar to the way *Acinetobacter baumannii*, also an environmental GNB, has come to be considered an opportunistic pathogen. It was the 12th most common BSI GNB isolate in a recent study from a public hospital in San Francisco (269).

Another Enterobacter spp., *E. absuriae* was the most common cause of UTI and PNA among the 13 saprophytic GNB species. The UTI was associated with prostate cancer in one patient (142), and patients with PNA had other underlying medical conditions, including cachexia and possible HIV infection (137). The Enterobacter spp. are environmental saprophytes. Why this particular Enterobacter spp. and not others are associated with UTI and PNA is not known. Furthermore, no infections were reported to be caused by 7 of the 20 saprophytic species (Table 1).

One major concern with these GNB organisms is their association with HCA infections. These organisms carry drug-resistance genes typically found in human primary GNB pathogens. In fact, two of these species—*E. aerogenes* and *Pantoea agglomerans* – carried the most common and globally distributed ESBL gene *bla*_CTX-M-15_, found in the most common pandemic ExPEC lineage ST131 (17, 270). Other studies have found drug-resistance genes harbored by bacterial pathogens in environmental saprophytic organisms (12, 271, 272). Although we found no evidence that such drug-resistance genes are transferred from these saprophytes to GNB pathogens, they appear to be more ubiquitous across saprophytes than previously recognized.

There are several limitations to this type of bibliographic search. Quantitative comparison of the GNB species and their frequency of infections and associations with comorbidities or medical interventions could not be accurately performed, since not all reports included comparable data. Systematic or population-based surveys of these GNB organisms have not been conducted in many places, so our summary of the frequencies of the species and their association with infections could have been biased by a small number of reports. Nevertheless, the search has shown that these saprophytic GNB do cause infections, particularly in people with comorbidities, and that they harbor a large repertoire of drug-resistance genes.

*Acinetobacter baumannii*, which first began to appear in hospitals worldwide in the 1970s (273, 274), has recently become the most common non-fermentative HCA pathogen in many hospitals in Latin America, surpassing *P. aeruginosa* (3). In the United States, it accounts for about 3% of HCA infections, and is currently the second most common non-fermentative organism after *P. aeruginosa* in most hospitals (2). GNB have been a frequent cause of HCA infections for many decades, but the proportion of such infections caused by multidrug-resistant strains and new species have steadily increased in the last 20 years worldwide. It is possible that the same factors that facilitated the first appearance and subsequent endemicity of *A. baumannii* in healthcare institutions could contribute to the introduction and establishment of other environmental saprophytic GNB into hospitals. With ever-expanding use of immunosuppressive drugs and biologics, invasive procedures, and increased prevalence of patients with chronic medical conditions as well as advanced age, these saprophytic GNB infections are likely to increase. Hospitals need to be prepared to detect these organisms, especially in infections in which recognized pathogens are not recovered. This work also sheds light on the role of antibiotics used in animal husbandry. Such use can select for antimicrobial drug resistance in commensal bacteria in animal intestines as well as in saprophytes in the environment with ramifications for human health as demonstrated by this study.

## Author Contributions

LR conceived the idea for this systematic review, based on previous work performed by ER. ER conducted the literature review, selected the publications for inclusion in the final analyses, and analyzed the data together with LR. ER wrote the first draft, which was reviewed by LR, and both authors reviewed multiple drafts to write the final draft.

## Conflict of Interest Statement

The authors declare that the research was conducted in the absence of any commercial or financial relationships that could be construed as a potential conflict of interest.
