# Supplementary material for: Infections Caused by Antimicrobial Drug-Resistant Saprophytic Gram-Negative Bacteria in the Environment
Source: Front Med (Lausanne). 2017 Oct 30;4:183. doi: 10.3389/fmed.2017.00183 (PMC5670356; doi:10.3389/fmed.2017.00183)
Supplement: Supplementary file 1 [file Table_1.DOCX]

Supplementary table 1. Co-morbidities, healthcare interventions, and inciting events associated with infections caused by saprophytic GNB species.

| **Organism** | **Infection** | **Co-morbidities (number of cases)** | **Associated medical procedures** | **Associated inciting event** | **References** |
| --- | --- | --- | --- | --- | --- |
| Enterobacter aerogenes | BSI | acute myelogenous leukaemia (2), acute myocardial infarction (3), disseminated liver cancer (1), HBV (1), liver transplant (1), gastric cancer (1), cancer (31), biliary disease (21), diabetes (10), liver cirrhosis (1), transplantation (1), end-stage renal disease (1), malignancies, hypertension, diabetes, chronic renal disease, stroke, congestive heart failure, liver cirrhosis, acute pancreatitis with biliary tract obstruction, choledocholithiasis, autoimmune disease, COPD, polymicrobial infection, neutropenia (1), chemotherapy (1), cancers, ARDS (10), premature birth (10), smoking, etoh, cardiomyopathy, PE, vena cava filter, AAA, pseudomembranous colitis, retroperitoneal hematoma, ALL, chemo complication with perforation of the distal esophagus, severe esophageal stenosis and a tracheo-esophageal fistula, hemorrhoidectomy | Liver transplant (1), CABG (1), coronary resvascularization (1), endoscopic submucosal dissection for gastric neoplasia (1), nosocomial (49), operations within 14 days of bacteremia, invasive procedure, chemotherapy, immunosuppressive drugs, endovascular stent graft, esophagectomy and insertion of a gastric tube, port-a-cath, perforation of colon, |  | Aschbacher 2010, Kasap 2010, Chen H 2009, Diene 2012, Torres 2014, Kato 2012, Song 2010, Chang 2009, Souli 2008. Huh 2013, Narayan 2009, Ghosh 2006, Van der Meer 2006, Parker 2010 |
|  | UTI | DM, HTN, IHD, DLP, COPD, Hypothyroid, ESRD, HIV, TB, Fanconi syndrome, renal failure | Foley catheter |  | Taneja 2008, Abukabar 2013, Bilgen 2006, Cunha 2007, Ondounda 2011 |
|  | PSI |  | penile prosthesis placement, neurosurgery, placement of ventricular external drainage, CSF cranial fistual |  | Kava 2011, Laguna-del-Estal 2010 |
|  | SSTI | obese (1), trauma patient (1), hemorrhagic shock (1), DM (1), dermatomyositis with calcifications, on immunosuppressant treatment, DM, ESRD, diabetic foot ulcer, renal dialysis, continuous cycling peritoneal dialysis | transmetatarsal amputation |  | Utrup 2010, Lee 2011, Corea 2011, Iwalokun 2011, Edouard 2010, Van Ende 2010 |
|  | Osteomyelitis | diabetes (1), obese (1), trauma patient (1), hemorrhagic shock (1) |  |  | Lin 2010, Utrup 2010, Lee 2011 |
|  | PNA | malaria, AKI, HD |  |  | Santana 2006 |
|  | Others (peritonitis, endophthalmitis, meningitis, pyelonephritis, optic neuropathy/mucocele, Spontaneous infectious aortitis, pyogenic liver abscess) | renal transplant with ADPKD (1), chronic renal allograft dysfunction (1), immunosuppression (1), fungal sinusitis (1), mucormycosis orbital cellulitis (1), smoking, etoh, cardiomyopathy, PE, vena cava filter, AAA, pseudomembranous colitis, retroperitoneal hematoma, HTN, HIV, TB, Fanconi syndrome, renal failure, cerebral palsy, CAD, PVD, CABG, iliac angioplasty, DM, hemorrhoidectomy | cataract surgery, endovascular stent graft, neurosurgery, placement of ventricular external drainage, CSF cranial fistual, intrathecal blacofen |  | Rodriges 2012, Zanation 2013, Bhat 2014, Ghosh 2006, Laguna-del-Estal 2010, Ondounda 2011, Wunderlich 2006, Smith 2007, Rondina 2006, Parker 2010 |
| Enterobacter amnigenus | BSI | heart transplant, CHF, CABG, mild rejection of transplant, steroids, myelodysplasia | steroids, CVC, blood transfusion |  | Sheng 2012, Bollet 1991, Jan 1983 |
|  | SSTI |  |  | MVA | Corti 2009 |
|  | Others (exogenous endophthalmitis: unliateral hypopyon uveitis, endophthalmitis) | DM, HTN, Hansen's disease |  |  | Westerfeld 2009, Korah 2007 |
| Enterobacter asburiae | BSI | immunocompromised, cancer, non-fatal SIDS, AAA |  |  | Kang 2004, Novais 2012, Huh 2013, Brenner 1986 |
|  | UTI | prostate cancer |  |  | Aibinu 2012, Brenner 1986 |
|  | PSI |  | C-section, small bowel resection, colostomy, cholecystectomy |  | Brenner 1986 |
|  | SSTI |  | wound, boil, abscess | all terrain vehicle crash | Brenner 1986, Koth 2012 |
|  | PNA | cachexia, oral thrush, bilateral axillary and clavicular lymphadenopathy, and generally clear lungs, suspected HIV+ |  |  | Stewart 2001, Brenner 1986 |
|  | Others |  |  |  | Brenner 1986 |
| Enterobacter kobei | BSI | vaginal cancer | colpectomy, hysterectomy, and bilateral adenectomy with complete lymphadenectomy of the lower pelvis |  | Hoffmann 2005 |
|  | UTI | vaginal cancer | colpectomy, hysterectomy, and bilateral adenectomy with complete lymphadenectomy of the lower pelvis |  | Hoffmann 2005 |
|  | PSI | vaginal cancer | colpectomy, hysterectomy, and bilateral adenectomy with complete lymphadenectomy of the lower pelvis |  | Hoffmann 2005 |
| Enterobacter ludwigii | UTI |  |  |  | Hoffmann 2005 |
|  | PSI | aneurysmal bone cyst in left distal femur |  |  | Khajuria 2013 |
|  | Others (throat) |  |  |  | Hoffmann 2005 |
| Erwinia persicina | UTI | CAD, CHF, HTN, DM, lower leg hematoma, stasis dermatitis |  |  | O'Hara 1998 |
| Pantoea agglomerans | BSI | Dm (2), HCV (1), liver cancer, hepatic encephalopathy (1), liver transplant (1), C. difficile colitis (1), immonusuppressive therapy (2), con-committant rotavirus infection (1), prolonged rupture of membranes (2), chorioamnionitis (1), preterm neonates (5), respiratory distress (2) jaundice (1), pre- necrotizing enterocolitis (1), lymphoma (1), congenital heart disease (1), pulmonary atresia with intact ventricular septum (1), DIC (1), Hirschsprung disease (1), laparotomy (1), short bowel syndrome (1), HTN (1), steroid-induced hyperglycemia (1), acute myeloid leukemia (1), haemophilia A with factor VIII activity of 1% (1), lymphoblastic leukemia during induction therapy (1), diabetic nephropathy (1), cystic fibrosis, neurological impairment, tumor, intestinal malabsorption, hematological malignancies, CHD, necrotizing colitis, thrombotic thrombocytopenic purpura (2), Guillain-Barre syndrome (1), Congestive heart failure (2), lung transplant (2), colon cancer, prostate cancer, chemotherapy, neurofibromatosis, pelvic neurofibrosarcoma which relapsed, radiation therapy and surgery, ESRD | intubation and mechanical respiration (5), CPAP (5), chest tube (1), CVC (28), CPAP, parenteral nutrition, port-a-cath with ITI therapy with rFVIII concentrate (1), port-a-cath (1), continuous cycling peritoneal dyalisis (1), contaminated anticoagulant citrateedextrose, hemodyalisis, plasmapheresis, IJ catheter, cuffed catheter hemodialysis | Hickman line contaminatd with water from botanical garden | Morales-Ruiz 2010, Cicchetti 2006, Lalas 2010, Aly 2008, Russell 2007, Bergman 2007, Hsieh 2007, Uche 2008, Grangl 2013, Fernandez-Munoz 2014, Kahveci 2011, Cruz 2007, Markovska 2014, aibinu 2012, Liberto 2009, Bicudo 2007, Boszczowski 2011, Christakis 2007, Marcos Sanchez 2006, Wong 2012 |
|  | UTI | cystic fibrosis, neurological impairment, tumor, intestinal malabsorption, hematological malignancies, CHD, necrotizing colitis |  |  | Cruz 2007 |
|  | SSTI | cystic fibrosis, neurological impairment, tumor, intestinal malabsorption, hematological malignancies, CHD, necrotizing colitis |  | puncture wound in foot, foreign body in wound: wood splinter, date palm thorn | Harris 2010, Deps 2006, Cruz 2007, Vaiman 2013 |
|  | Osteomyelitis | sickle cell (1), mechanical aortic valve (1), cystic fibrosis, neurological impairment, tumor, intestinal malabsorption, hematological malignancies, CHD, necrotizing colitis | closed fracture |  | Bachmeyer 2007, Cruz 2007, Labianca 2013 |
|  | PNA | diabetes mellitus, HCV, liver cancer, hepatic encefalopathy, liver transplant, C. difficile colitis, immonusuppressive therapy, tetralogy of Fallot (1) | en-bloc heart and lung transplantation (1) |  | Morales-Ruiz 2010, Shubov 2011 |
|  | Others (peritonitis, septic arthritis, endophthalmitis, liver abscess, prosthetic joint infections, endocarditis, knee laceration, brain abscess, laryngitis, endocarditis, pyogenic liver abscess) | multiple myeloma (1), chronic kidney disease (1), nephropathy (1), ESRD (6), FSGS (1), chronic ambulatory peritoneal dialysis (1), depression (1), duodenal ulcers (1), prior episodes of peritonitis (1), ILD (1), corticosteroid therapy (1), HTN (3), OA (1), congenital hydronephrosis (1), benign nephroangiosclerosis (1), diabetes, obesity (2), ischemic cardiomyopathy (1), obstructive uropathy (1), hyperethrysosis (1), DM(1), diabetic nephropathy (2), cystic fibrosis, neurological impairment, tumor, intestinal malabsorption, hematological malignancies, CHD, necrotizing colitis, MRSA osteomyelitis, BSI, appendectomy | continuous cycling peritoneal dyalisis (7), IJ catheter, cuffed catheter hemodialysis | plant product puncture (9) (rose thorn, date palm tree thorn, wood splinters, lemon tree thorn, wood fence splinter), contamination of catheter with nonsterile surface, total hip arthroplasty, worked in garden | Borras 2009, Duerinckx 2008, Lim 2006, Seok 2010, Fullerton 2007, Magnette 2008, Rave 2012, Ferrantino 2008, Habhab 2008, Kazancioglu 2014, Kletke 2014, Hischebeth 2013, Kahveci 2011, Volksche 2009, Cruz 2007, Chen 2013, Moreiras-Plaza 2009, Wong 2012, Santos Rodrigues 2009 |
| Pantoea ananatis | BSI | DM, CVA, DVT, lower GI hemorrhage | colonoscopy |  |  |
|  | Others (corneal infiltrate, undifferrentiated and reactive arthritis) | sexually acquired reactive arthritis |  | ocular trauma with rice husk | Manoharan 2012, Siala 2008 |
| Pseudomonas putida | BSI | gastric cancer, sigmoid colon adenocarcinoma, liver cirrhosis with HCV and hepatocellular carcinoma, Burkitt lymphoma, liver cirrhosis due to HBV and hepatocellular carcinoma, common variable immunodeficiency after developing autoimmune hemolytic anemia, hypertension, DM (2), stroke, COPD, lymphoproliferative syndrome, CHF, oesophageal perforation, CT surgery, chronic renal insufficiency, PVD, chronic nonhealing LE ulcer, Burkitt's lymphoma, common-types acute lymphoblastic leukemia, acute fungal sinusitis, BSI due to S. epidermidis, immunocompromise, neutropenia (3), ALL (3), traumatic intracranial hemorrhage, bacterial meningitis, esophageal cancer, ectopic pregancy, traumatic lung contusion, liver cirrhosis, variceal bleed, gastric cancer, ischemic heart disease, cardiac arrest, HTN, SMA infarction, pleomorphic leiomyosacroma, cholangiosarcoma, lung cancer, acute alcoholc hepatitis, neonates, solid tumors (32), hematologic malignancies (7), myasthenia gravis (1), retinoblastoma, hepatoblastoma, acute myeloblastic leukaemia, necrotizing fasciitis | gastrectomy (2), colectomy, CVC (40), liver transplantation, with indewelling biliary drainage tube, percutaneous transhepatic gallbladder drainage, chemotherapy (5), autologous bone marrow transplantation (2), sternotomy, urinary catheter, endotracheal tube, chest tube, peritoneal drainage, biliary stent, esophagectomy, small bowel resection, total colectomy, RUL lobectomy, ICU (intubation, CVC...), contaminated catheter lock infused with heparin (32), short-term intravascular device (5), Hickman catheter, mechanical ventilation, umbilical catheterization | MVA (1) | Yoshino 2011, Treviño 2010, Poirel 2009, Thomas 2013, Almeida 2012, Rokicka 2006, Shah 2007, Mitsuda 2011, Kim 2012, Oguz 2010, Souza Dias 2008, Aumeran 2007, Liu 2014, Carinder 2010, Erol 2013 |
|  | UTI | bladder cancer, gastric cancer | urostomy, gastrectomy, urine catheter (2) |  | Treviño 2010, Almuzara 2007, Kim 2012 |
|  | PSI | gastric cancer, sigmoid colon adenocarcinoma, liver cirrhosis with HCV and hepatocellular carcinoma, bladder cancer, ESRD, automated peritoneal dialysis, ALL, esophageal cancer, gastric cancer, SMA infarction, lung cancer | Gastrectomy, colectomy, liver transplant, urostomy, bilateral transtibial amputations, laparoscopic removal of ovarian cyst, CT surgery, autologous bone marrow transplantation, sternotomy, esophagectomy, gastrectomy, small bowel resection, total colectomy, RUL lobectomy | blast injuries, MVA with esophageal perforation | Yoshino 2011, Treviño 2010, Carpenter 2008, Dervisoglu 2008, Poirel 2009, Rokicka 2006, Shah 2007, Kim 2012 |
|  | SSTI | bladder cancer, chronic renal insufficiency, PVD, chronic nonhealing LE ulcer, pleomorphic leiomyosarcoma, neutropenia | urostomy, bilateral transtibial amputations | blast injuries | Treviño 2010, Carpenter 2008, Thomas 2013, Kim 2012 |
|  | PNA | common variable immunodeficiency after developing autoimmune hemolytic anemia, esophageal cancer, cardiac arrest, IHD, HTN, DM, lung cancer, traumatic ICH, acute alcoholic hepatitis | percutaneous thranshepatic gallbladder drainage, esophagectomy, RUL resection, ventilator (4) |  | Yoshino 2011, Almuzara 2007, Kim 2012 |
|  | Others (cholangitis, cholecystitis, peritonitis, meningitis, bilateral recurrent conjunctivitis and chalazion, keratitis) | liver cirrhosis due to HBV and hepatocellular carcinoma, common variable immunodeficiency after developing autoimmune hemolytic anemia, ESRD, automated peritoneal dialysis, corneal ulcer | liver transplantation, with indewelling biliary drainage tube, pneumonia, percutaneous thranshepatic gallbladder drainage, lapaoscopic removal of ovarian cyst complicated by subsequent peritonitis, orthokeratology | fall | Yoshino 2011, Dervisoglu 2008, Toru 2008, Zuberbuhler 2012, Ying-Chen 2006 |
| Rahnella aquatilis | BSI | oesophagus and stomach lesions caused by the accidental ingestion of a caustic product, renal carcinoma with colonic and pancreatic metastases, HIV, IVDU, HBC, HCV, HDV, pneumonia, stage IV neuroblastoma with disseminated metastases, chemotherapy and autologous transplantation with peripheral stem cells, BPH, sickle cell disease, transfusion-associated hemochromatosis, juvenile rheumatoid arthritis, pancreatic cancer, ALL, acute lymphoblastic leukaemia, DM2, asthma | CVC (5), parenteral nutrition (2), total nephrectomy with pancreatectomy and colonic resection, chemotherapy | IV injection of contaminated D5W+Vit B complex outside hospital | Caroff 1998, Funke 1995, Hoppe 1993, Tash 2005, Gaitan 2010, Chang 1999, Liberto 2009, Carinder 2010, Goubeau 1988 |
|  | UTI | ESRD, HTN, DM, renal transplant, chemotherapy, S. aureus arthritis in wrist, miliary TB, UTI by K. pneumoniae, BPH | renal transplant |  | Alballaa 1992, Tash 2005 |
|  | PSI | renal carcinoma with colonic and pancreatic metastases, osteoporosis, laryngeal cancer, DM, hypercholesterolemia | parenteral nutrition, total nephrectomy with pancreatectomy and colonic resection, internal osteosynthesis, tracheostomy |  | Caroff 1998, Maraki 1994 |
|  | Others (acute gastroenteritis, infective endocarditis) | AIDS, ASD, pulmonary valve stenosis |  |  | Reina 1996, Matsukura 1996 |
| Rhizobium spp | BSI | SCID, chemotherapy-refractory adult T-cell lymphoma, diabetic nephropathy, ESRD, stage IV ovarian carcinoma, peritoneal carcinomatosis, epilepsy, stroke, gastric hemorrhage, chronic renal failure, and bladder catheter for prostate hypertrophy, ARF, osteosarcoma with metastases in the lungs and bones, corticosteroid therapy, DM (2), stage III-A IgG kappa multiple myeloma undergoing autologous stem cell mobilisation, prematurity, bilateral pneumothorax, pulmonary hypertension, pulmonary interstitial emphysematous changes with bronchopulmonary dysplasia, necrotizing enterocolitis, Acute MI, CAD, HTN (2), melena, CABG, squamous cell carcinoma of the lung, chemotherapy, ALL (2), asthma | CVC (7), hematopoetic stem cell transplantation, allogenic bone marrow transplantation, tunneled-cuffed hemodialysis catheter, CABG, splenectomy, hepatorrhapy, tracheostomy, percutaneous angioplasty, percutaneous endoscopic gastrostomy, multiple orthopedic procedures, mechanical ventilation, chemotherapy | contamination of CVC line due to exposure to soil, MVA (splenic and liver lacerations, left kidney infarction, right internal carotid artery pseudoaneurysm, degloving injury and multiple fractures of the left hand, left radial and ulnar shaft fractures, left lower extremity open ankle fracture, comminuted public symphysis and sacral fractures, bilateral occipital condylar fractures, sternal fracture, pneumomediastinum, bilateral, occipital condylar fractures, sternal fracture, pneumomediastinum, bilateral pneumothoracies, and multiple rib fractures) | Cipe 2010, Kato 2009, Hanada 2009, Detrait 2008, Christakis 2006, Chen 2008, Sood 2010, Ponnapula 2013, Kaseltiz 2012, Karadag 2013, Mantakatis 2010 |
|  | PSI | Acute MI, CAD, DM, HTN, melena, CABG, OSA, HTN, DM2, long-term steroid treatment | CABG, cataract surgery (3) |  | Sood 2010, Moreau-Gaudry 2012 |
|  | Osteomyelitis | DM, peripheral neuropathy |  |  | Dhatariya 2011 |
|  | Others (cholecystitis, cerebral abscess, endophthalmitis, peritonitis, endocarditis of native mitral valve, septic emboli, endocarditis) | epilepsy, stroke, gastric hemorrhage, chronic renal failure, and bladder catheter for prostate hypertrophy, ARF, AIDS, OSA, HTN, DM2, long-term steroid treatment, immunoglobulin A nephropathy, distant right mastectomy for adenocarcinoma, ESRD stage 5 on HD | cataract surgery (3), continuous ambulatory peritoneal dialysis, CVC, permacath | perforated duodenal ulcer | Detrait 2008, Luis 2012, Moreau-Gaudry 2012, Tsai 2013, Chao 2014, Guerra 2013, Pinera Gonsalves 2013 |
| Serratia fonticola | BSI | MVA with orthopedic procedure, obesity | MVA |  | Bollet 1991 |
|  | SSTI | MVA with orthopedic procedure, obesity | MVA | bear bite | Bollet 1991, Kunimoto 2004, Pfyffer 1992 |
|  | Others (spetic arthritis) |  |  | thorn penetration | Gorret 2009 |
| Serratia proteamaculans | PNA | immunocompromised, dental abscess, PUD, cholecystitis, chronic calcifying pancreatitis, cirrhosis with steatosis | tracheostomy, exploratory laparotomy, cholecystectomy |  | Bollet 1993 |
|  | Others | failed knee prosthesis |  |  | Temoin 2012 |
